# Supplementary material for: HPRT1 Promotes Chemoresistance in Oral Squamous Cell Carcinoma via Activating MMP1/PI3K/Akt Signaling Pathway
Source: Cancers (Basel). 2022 Feb 9;14(4):855. doi: 10.3390/cancers14040855 (PMC8870334; doi:10.3390/cancers14040855)

## Table S1. Primer sequences used in this study

| Primer name | Sequences |
|---|---|
| **HPRT1** | forward: 5 ′-GATCTGTGAGTTCATCAGCGGC-3′; |
| | reverse: 5 ′TGACGAAGCAGTCGTTGAGCGA-3′ |
| **GAPDH** | forward:5′-TGCACCACCAACTGCTTAG-3′ |
| | reverse: 5′-GATGCAGGGATGATGTTC-3′ |
| **MMP1** | forward: 5'-GGGGCTTTGATGTACCCTAGC-3' |
| | reverse: 5'-TGTCACACGCTTTTGGGGTTT-3' |
| **Sh-HPRT1#1** | CCAGGTTATGACCTTGATTTA |
| **Sh-HPRT1#2** | CCAGACTTTGTTGGATTTGAA |
| **Si-HPRT1#1** | GUUUAUUCCUCAUGGACUA |
| **Si-HPRT1#2** | GACUGAACGUCUUGCUCGA |
| **Si-MMP1#1** | TGAAGATGAAAGGTGGACCAA |
| **Si-MMP1#2** | GTTTGTGGCTTATGGATTCAT |

## Table S2. Primary antibody used in this study

| Primary antibody name | Species | Manufacturer and location |
|---|---|---|
| **HPRT1** | Rabbit | Cell Signaling Technology, Boston, US |
| **MMP1** | Mouse | GenTex, Texas, US |
| **GAPDH** | Mouse | Sigma Aldrich, St. Louis, Missouri, US |
| **γ-H2AX** | Mouse | Cell Signaling Technology, Boston, US |
| **HFM1** | Rabbit | Sigma Aldrich, St. Louis, Missouri, US |
| **CDRT1** | Rabbit | Biorbyt Ltd. 5 Orwell Furlong, United Kingdom |
| **TECRP1** | Rabbit | Sigma Aldrich, St. Louis, Missouri, US |
| **FSCN2** | Rabbit | Abclonal Technology, Wuhan, China |
| **PI3K , p-PI3K** | Rabbit | Cell Signaling Technology, Boston, USA |
| **AKT, p-AKT** | Mouse | Sigma Aldrich, St. Louis, Missouri, US |
| **Survivin** | Mouse | Sigma Aldrich, St. Louis, Missouri, US |
| **C-myc** | Mouse | Cell Signaling Technology, Boston, USA |

**Table S3. Correlation between HPRT1 expression and clinicopathological characteristics of OSCC patients**

| Characteristics | HPRT1 expression | | | |
|---|---|---|---|---|
| | NO. | Low No. | High No. | P value |
| **Total No.** | 136 | 68 | 68 | |
| **Gender** | | | | 0.571 |
| Male | 86 | 45 | 43 | |
| Female | 50 | 23 | 25 | |
| **Age** | | | | 0.500 |
| ≤55 | 67 | 32 | 35 | |
| >55 | 69 | 36 | 33 | |
| **AJCC** | | | | 0.001 |
| StageI-II | 66 | 42 | 24 | |
| StageIII-IV | 70 | 26 | 44 | |
| **T classification** | | | | 0.003 |
| T1-T2 | 77 | 46 | 31 | |
| T3-T4 | 59 | 22 | 37 | |
| **N classification** | | | | 0.001 |
| N0 | 73 | 44 | 29 | |
| N+ | 63 | 24 | 39 | |
| **Smoking** | | | | 0.230 |
| Yes | 66 | 40 | 26 | |
| No | 70 | 28 | 42 | |
| **Drinking** | | | | 0.487 |
| Yes | 79 | 28 | 51 | |
| No | 57 | 40 | 17 | |
| **Relapse** | | | | 0.010 |
| Yes | 45 | 16 | 29 | |
| No | 91 | 52 | 39 | |

**Table S4. Univariate and multivariate analysis of factors associated with overall survival in OSCC patients**

| | Univariate analysis | | Multivariate analysis | |
|---|---|---|---|---|
| **Variate** | **HR (95% CI)** | **P value** | **HR (95% CI)** | **P value** |
| **Gender** | | | | |
| Male | 1(Ref) | | | |
| Female | 0.663(0.403, 1.029) | 0.107 | | |
| **Age** | | | | |
| ≤55 | 1(Ref) | | | |
| >55 | 1.288(0.809, 2.049) | 0.286 | | |
| **Drinking** | | | | |
| No | 1(Ref) | | | |
| Yes | 1.119(0.746, 1.903) | 0.464 | | |
| **Smoking** | | | | |
| No | 1(Ref) | | | |
| Yes | 1.247 (0.785, 1.981) | 0.349 | | |
| **N classification** | | | | |
| N0 | 1(Ref) | | 1(Ref) | |
| N+ | 1.780(1.117, 2.834) | 0.015 | 1.143(0.690, 1.895) | 0.603 |
| **T classification** | | | | |
| T1-T2 | 1(Ref) | | 1(Ref) | |
| T3-T4 | 2.055(1.292, 3.268) | 0.002 | 1.532(0.942, 2.492) | 0.086 |
| **AJCC Stage** | | | | |
| Stage I-II | 1(Ref) | | 1(Ref) | |
| Stage III-IV | 2.256(1.387, 3.669) | 0.001 | 0.897(0.897, 2.625) | 0.118 |
| **HPRT1 expression** | | | | |
| Low | 1(Ref) | | 1(Ref) | |
| High | 2.833(1.744, 4.602) | < 0.0001 | 2.118(1.240, 3.617) | 0.006 |

**Table S5. Univariate and multivariate analysis of factors associated with delapse-free survival in OSCC patients**

| Variate | Univariate analysis | | Multivariate analysis | |
|---|---|---|---|---|
| | HR (95% CI) | P value | HR (95% CI) | P value |
| **Gender** | | | | |
| Male | 1(Ref) | | | |
| Female | 0.661(0.402, 1.086) | 0.102 | | |
| **Age** | | | | |
| ≤55 | 1(Ref) | | | |
| >55 | 1.345(0.844, 2.141) | 0.212 | | |
| **Drinking** | | | | |
| No | 1(Ref) | | | |
| Yes | 1.114(0.698, 1.779) | 0.650 | | |
| **Smoking** | | | | |
| No | 1(Ref) | | | |
| Yes | 1.197 (0.754, 1.901) | 0.447 | | |
| **N classification** | | | | |
| N0 | 1(Ref) | | 1(Ref) | |
| N+ | 1.827(1.147, 2.912) | 0.011 | 1.082(0.664, 1.817) | 0.767 |
| **T classification** | | | | |
| T1-T2 | 1(Ref) | | 1(Ref) | |
| T3-T4 | 2.094(1.315, 3.334) | 0.020 | 1.515(0.928, 2.474) | 0.097 |
| **AJCC Stage** | | | | |
| Stage I-II | 1(Ref) | | 1(Ref) | |
| Stage III-IV | 2.288(1.405, 3.726) | 0.001 | 1.545(0.893, 2.674) | 0.120 |
| **HPRT1 expression** | | | | |
| Low | 1(Ref) | | 1(Ref) | |
| High | 3.101(1.898, 5.006) | < 0.0001 | 2.393(1.394, 4.108) | 0.002 |

Figure S1HPRT1 mRNA levels in OSCC tissues and OSCC cell lines.

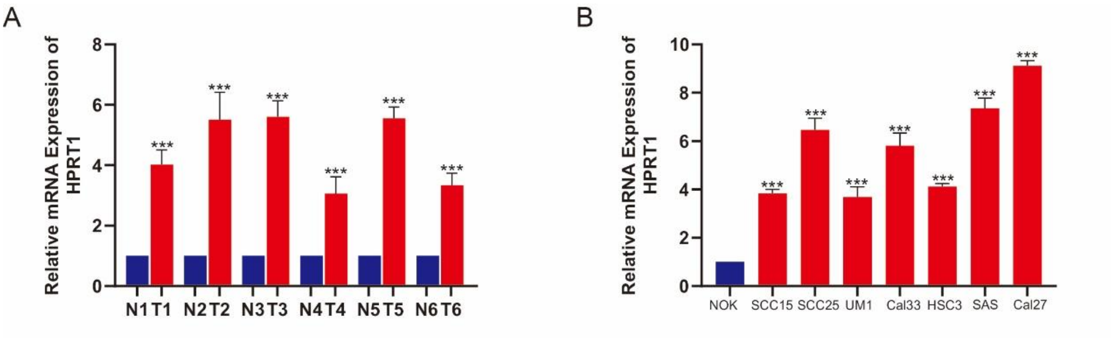


Figure S2 Original pictures of western blot bands in Figure 2.

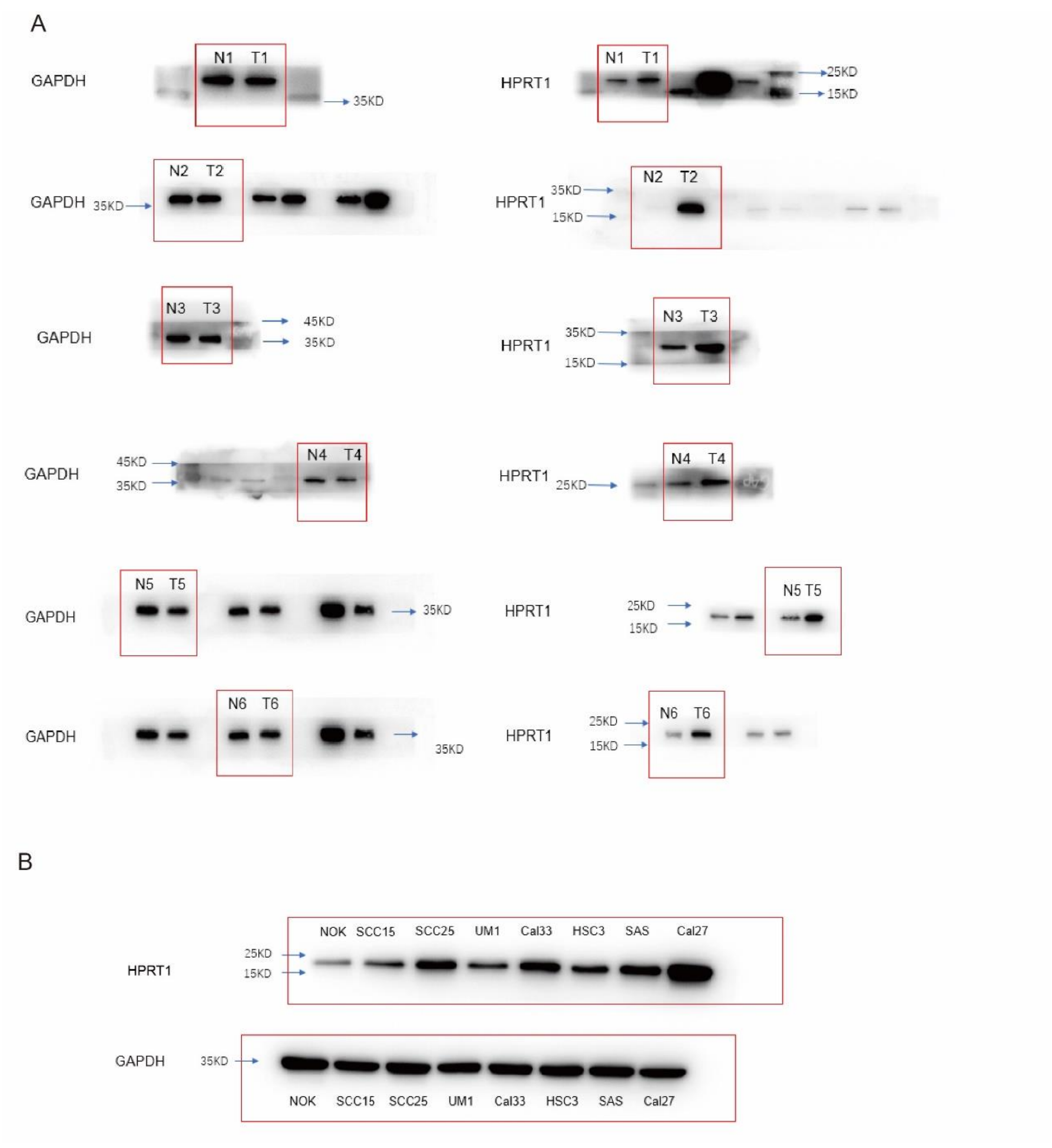

Figure S3 Original pictures of western blot bands in Figure 3.

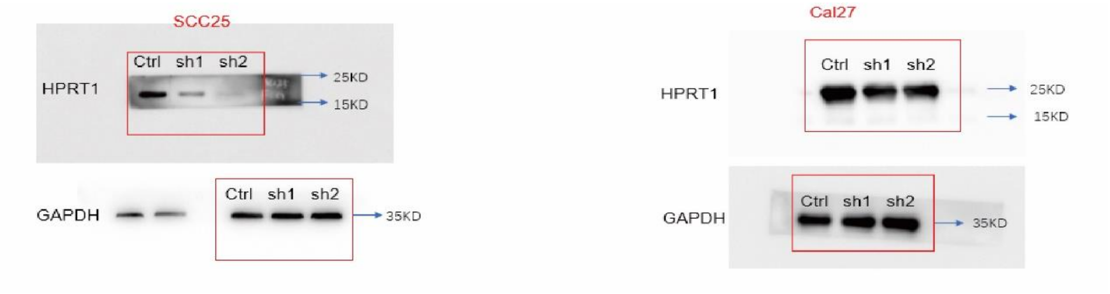


Figure S4 Original pictures of western blot bands in Figure 4.

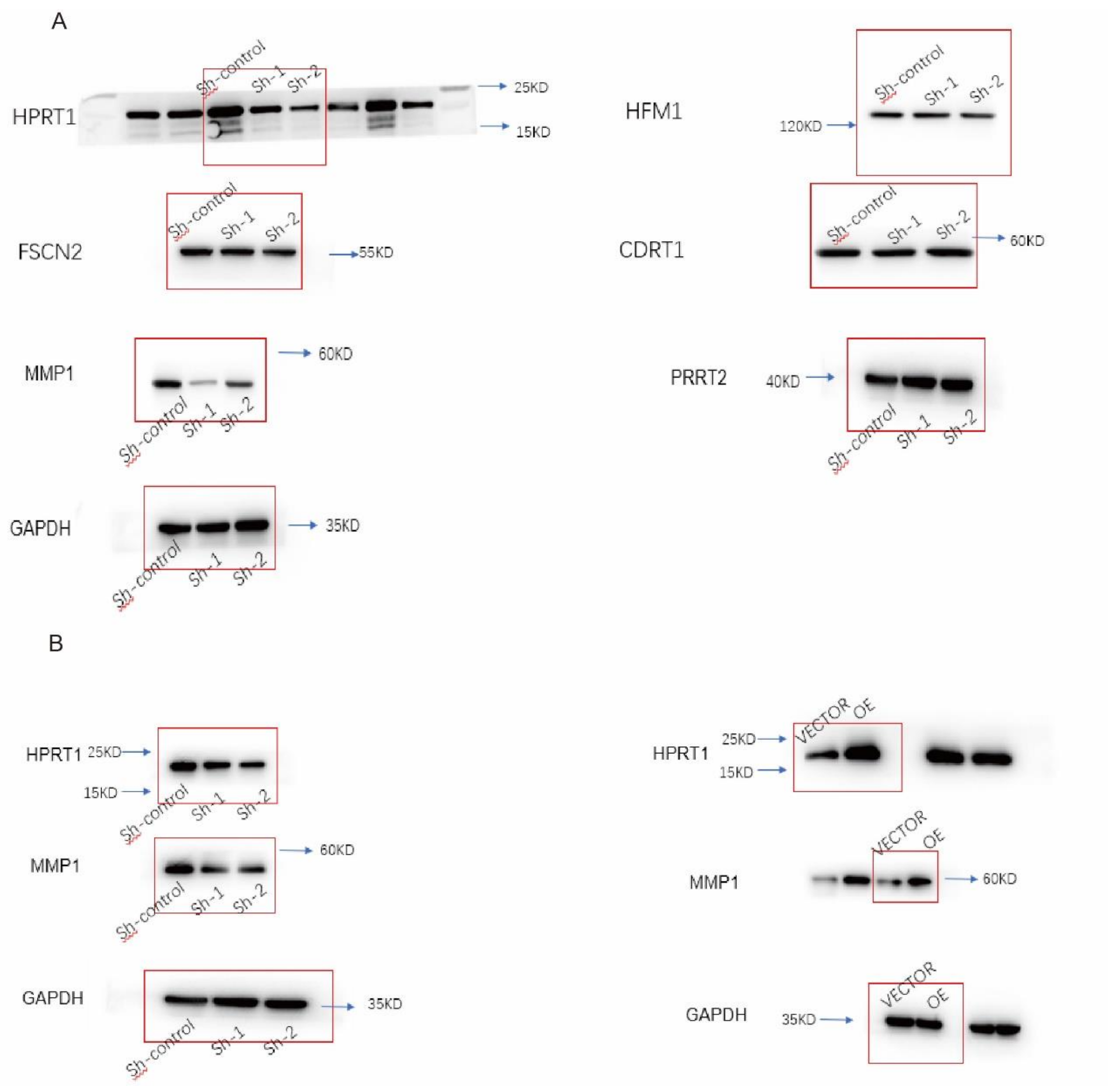

Figure S5 Original pictures of western blot bands in Figure 6.

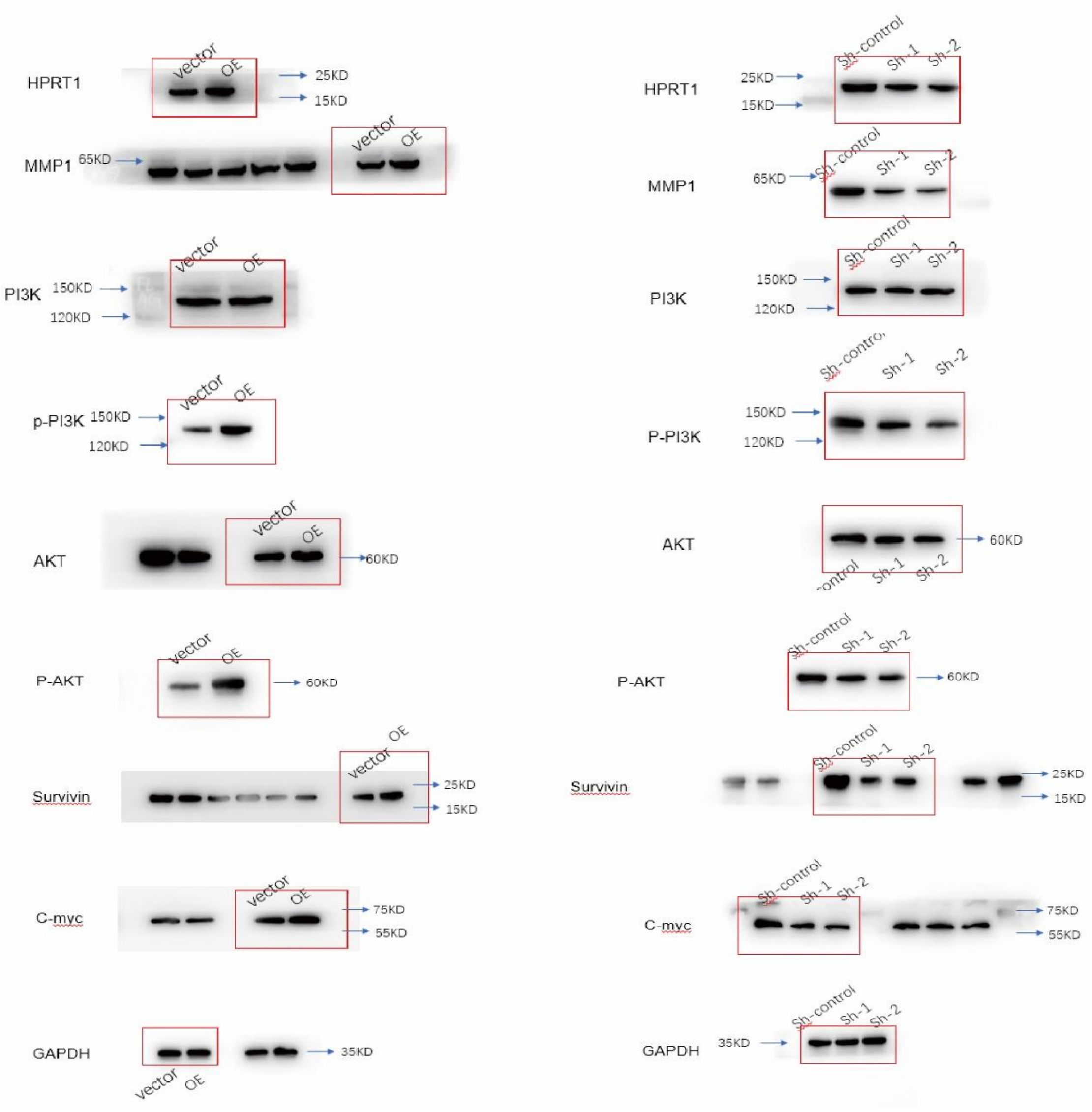

Supplement: Supplementary file 1 [file cancers-14-00855-s001.zip › cancers-1561954-supplementary.pdf]
